# Indirect calorimetry directed feeding and cycling in the older ICU population: a pilot randomised controlled trial

**DOI:** 10.1186/s12871-024-02507-8

**Published:** 2024-05-07

**Authors:** Ng Shu Hui Elizabeth, Tan Yanni, Leong Siaw May, Tiong Hui Fen, Li Xuanhui Janice, Kwan Peijun, Ong Sze Pheng, Toh Shi Jie, Loh Ne Hooi Will

**Affiliations:** Department of Anaesthesia, National University Hospital of Singapore, 5 Lower Kent Ridge Road, 119074 Singapore, Singapore

**Keywords:** Indirect calorimetry, Nutrition, Cycle ergometry

## Abstract

**Background:**

Older critically ill patients experience rapid muscle loss during stay in an intensive care unit (ICU) due to physiological stress and increased catabolism. This may lead to increased ICU length of stay, delayed weaning from ventilation and persistent functional limitations. We hypothesized that with optimal nutrition and early physical therapy acting in synergism, we can reduce muscle mass loss and improve functional outcomes.

**Methods:**

This was a prospective, single blinded randomized, controlled single-center pilot study to compare the lean muscle mass (measured at bilateral quadriceps femoris using ultrasound) of older ICU patients at 4 time points over 14 days between the control and intervention groups. The control group received standard weight-based empiric feeding and standard ICU physiotherapy. The intervention group received indirect calorimetry directed feeding adjusted daily and 60 min per day of cycle ergometry. 21 patients were recruited and randomized with 11 patients in the control arm and 10 patients in the intervention arm. Secondary outcome measures included ICU and hospital mortality, length of stay, functional assessments of mobility and assessment of strength.

**Results:**

Median age was 64 in the control group and 66 in the intervention group. Median calories achieved was 24.5 kcal/kg per day in the control group and 23.3 kcal/kg per day in the intervention group. Cycle ergometry was applied to patients in the intervention group for a median of 60 min a day and a patient had a median of 8.5 sessions in 14 days. Muscle mass decreased by a median of 4.7cm^2^ in the right quadriceps femoris in the control group and 1.8cm^2^ in the intervention group (*p* = 0.19), while the left quadriceps femoris decreased by 1.9cm^2^ in the control group and 0.1cm^2^ in the intervention group (*p* = 0.51).

**Conclusion:**

In this pilot study, we found a trend towards decrease muscle loss in bilateral quadriceps femoris with our combined interventions. However, it did not reach statistical significance likely due to small number of patients recruited in the study. However, we conclude that the intervention is feasible and potentially beneficial and may warrant a larger scale study to achieve statistical significance.

**Trial Registration:**

This study was registered on Clinicaltrials.gov on 30th May 2018 with identifier NCT03540732.

## Introduction

Critically ill patients experience rapid skeletal muscle loss during stay in an intensive care unit (ICU) due to physiological stress and increased catabolism [1]. This leads to increased ICU length of stay, delayed weaning from ventilation and other morbidities including persistent functional limitations [2, 3]. This is more so in the older patients in ICU due to pre-existing sarcopenia [4, 5], leading to the increase in mortality.

Currently, the international practice of using a weight based empiric formula to estimate nutritional and caloric requirement in the ICU may not be accurate for patients who are underweight, overweight or fluid overloaded [6]. The underfeeding of a critically ill patient may lead to an increase in mortality as delays in initiation of feeding cannot be compensated at a later date[7]. Indirect calorimetry is the gold standard for assessing individual energy requirements [6]. However, studies done on indirect calorimetry directed feeding have not shown a significant impact on functional outcome [8]. We postulate that the patients included in these studies may not have received adequate physical therapy. In the same vein, studies have shown that physical therapy in the critically ill improves functional outcome only marginally or showed no difference in global muscle strength [9]. The marginal result could be due to inadequate feeding of the patients studied. Therefore, we hypothesized that with optimal nutrition and early physical therapy acting in synergism, we can reduce muscle mass loss and improve functional outcomes. The aim of the study was to assess whether a combination of indirect calorimetry directed feeding and cycle ergometry reduced muscle wasting in the older ICU population, compared to empirical weight-based feeding and standard physiotherapy. With increasing survival from ICU, we hope to reduce the burden of disability among the ICU survivors and contribute towards the reduction of post intensive care syndrome. This would help to return these patients to meaningful productive lives in the society.

## Methods

### Ethics

#### Ethics approval

was obtained from the National Healthcare Group Domain Specific Review Board (DSRB), Singapore. Informed written consent was obtained from the participant’s next of kin (main spokesperson), for all study participants. This study was registered with ClinicalTrials.gov on 30/05/2018 with the Protocol Record 2017/00573.

This was a prospective, single blinded, controlled single-centre pilot study comparing the lean muscle mass of older, critically ill patients admitted in the surgical intensive care unit (SICU) over 14 days between the control and intervention groups.

### Settings and participants

The study was conducted between 1st November 2017 and 31st June 2019 in the surgical ICU (SICU) at an 1000 bedded university academic centre. Patients were recruited by 3 study investigators independently adhering to inclusion and exclusion criteria (Table 1). The eligible patients’ next-of-kin (main spokesperson) were approached for a written consent. Decision was made to take consent from the next-of-kin as patients admitted to the ICU are generally critically ill, sedated and unable to communicate.

**Table 1:** Study inclusion and exclusion criteria

Inclusion Criteria	Exclusion Criteria
1. At least 50 years old 2. Mechanically ventilated within 3 days of ICU admission 3. Expected to be mechanically ventilated for more than 3 days at time of recruitment 4. Able to ambulate with or without a gait aid before hospitalization 5. Able to be enterally fed within 48 h of ICU admission	1. Unable to follow commands at baseline before hospital admission (e.g. Severe dementia) 2. Acute condition where cycling is a contraindication (e.g. leg fracture) 3. Not expected to survive the subsequent 48 h 4. Body habitus unable to fit the cycle ergometry 5. Patients at high risk of refeeding (i.e. NUTRIC score > = 5) : malnourished patients with anorexia nervosa, chronic malabsorption syndromes, chronic alcoholism, or patients with massive weight loss. 6. Extremes of BMI: i.e. BMI < 16 or > 30 7. Liver failure 8. Cycling exemptions precluding cycling within the first 4 days of mechanical ventilation 9. Requirement for inspired oxygen content (FiO2) greater than 0.8 10. Expected to be on renal replacement therapy for longer than 12 h per session 11. PEEP > 15mmHg 12. Air leaks through chest drains 13. Palliative goals of care or limitation of treatment established by the CARE form 14. Readmissions to ICU

We included patients who were at least 50 years old, expected to be intubated for at least 3 days, able to be enterally fed within 48 h of admission and who were able to ambulate with or without a gait aid before hospitalization. We excluded patients who were unable to follow commands at baseline before hospital admission, who were not expected to survive the following 48 h, who had conditions for which cycling was contraindicated (Eg. lower limb fractures), who were at high risk of refeeding syndrome, patients with liver failure or requiring renal replacement therapy, who required an inspired oxygen content of at least 80% or PEEP of more than 15, who had air leaks through chest drains and who had palliative goals of care.

After informed written consent taken by study team members, the patient was randomized by an assigned team member using a randomization program on www.sealedenvelope.com to either the control or intervention group in a 1:1 intervention allocation ratio and assessment of the primary outcome measure was done by an assessor blinded to the study intervention.

### Sample size

As sample size of 25 patients in each arm of the study was determined a priori to be appropriate to assess significant muscle loss of rectus femoris for a power calculation of 80% based on a previous ultrasound study by Seymour et al. [13].

### Control

The control group received standard weight-based empiric feeding of 25 kcal per kg per day based on their actual body weight if the body mass index (BMI) is below 30. If the BMI is above 30, the ideal weight is used. They also receive standard ICU physiotherapy as prescribed and at the discretion of their primary ICU team.

### Intervention

The intervention group received indirect calorimetry directed protocolised feeding regime adjusted daily, together with 60 min per day of cycle ergometry (MOTOmed letto2, RECK-Technik) each weekday. Patients underwent daily indirect calorimetry while intubated to obtain their resting energy expenditure (REE) and had their enteral feeds matched to this. Daily titration of feeds was carried out by investigators from the study team. Cycle ergometry was applied by investigators from the study team who reviewed a set of temporary cycling exemptions prior to commencing cycling. These included respiratory insufficiency with persistent oxygen saturation less than 88%, haemodynamic instability in patients with active myocardial ischemia, unstable or uncontrolled arrhythmia, increasing trend of vasoactive infusion, mean arterial pressure less than 60 mmHg or more than 110 mmHg, heart rate less than 40 or more than 140 beats per minutes. Uncontrolled pain, severe agitation, existing neuromuscular blockade, changes of goals of therapy to palliation as well as ICU team’s objection to cycling were also taken into account. A set of criteria for premature termination of cycle ergometry was also followed which include unplanned extubation, physiological derangements and either patient or physician’s request to stop intervention. The cycle ergometry was set to allow patients to participate in active cycling while providing an option of passive cycling to those who were not able to. Due to the nature of the intervention, the patient and primary ICU doctor were unable to be blinded. In the both groups, standardized early rehabilitation physical therapy was applied each weekday from randomization to ICU discharge.

### Variable and outcome measures

Demographic data (age, sex, weight, height, race, pre-existing conditions, diagnosis, APACHE II score, ICU and hospital length of stay (LOS), and survival) were collected for all patients from the electronic medical record.

The primary outcome was change in cross-sectional area of bilateral rectus femoris muscle as measured by ultrasound 14 days after recruitment into the study. An independent, blinded study investigator conducted an initial ultrasound measurement of bilateral quadriceps femoris cross sectional area of all patients within 24 h of recruitment. For the intervention group, the measurements were taken either before or within 2 h of starting cycle ergometry. Thereafter, an additional 3 measurements were taken over the next 14 days for all recruited patients. Cross-sectional area of the rectus femoris was measured by B-mode ultrasonography using an 8 MHz 5.6 cm linear transducer array (Sonosite, USA) as described by de Bruin et al. [13, 14]. The transducer was placed perpendicular to the long axis of the thigh on its superior aspect, three-fifths of the distance from the anterior superior iliac spine to the superior patellar border. Imaging was conducted supine with the rested leg supported in passive extension. Excess contact gel was applied to minimise underlying soft tissue distortion. Scanning depth was set to where the femur could be discerned for orientation. The cross-sectional area was calculated by a planimetric technique (Nemio, Toshiba Medical system) after the inner echogenic line of the rectus femoris was outlined by a movable cursor on a frozen image. The cross-sectional area was taken as an average of three consecutive measurements, within 10%.

Secondary outcomes evaluated potential benefits of the intervention. These included bedside functional outcome measurements, which consist of and Medical Research Council Sum Score (MRC-SS), Chelsea Critical Care Physical Assessment Tool (CPax), grip strength, quadricep strength were obtained at ICU discharge, at day 14 of the study and at hospital discharge. ICU, hospital length of stay and mortality rates were also obtained.

Grip strength was measured using the Jamar hydraulic hand dynamometer and was recorded in KgForce (KgF). Subjects were placed in a seated position with the tested limb supported on a firm surface with zero degree of shoulder flexion and 90 degrees of elbow flexion. They were then instructed to grip on the hand dynamometer was hard as possible. Quadriceps strength was measured using the Baseline push / pull dynamometer (Model 12–0342) and the recorded in KgForce (KgF). Subjects were placed in a supine position with the tested knee placed in 30 degrees knee flexion and the dynamometer was positioned proximal to the ankle malleoli [15]. Subjects were instructed to extend their knees against the dynamometer maximally.

### Statistical analysis

Statistical analysis was done using IBM SPSS Statistics for Windows, Version 25.0. Amonk, NY.

Analysis was intention to treat. Mann-Whitney U test was used for analysis of change in rectus femoris cross sectional area while continuous variables were analysed using the student T test. P value < 0.05 was considered as significant.

## Results

A total of 21 patients were recruited, of which 11 were in the control group and 10 in the intervention group.

Their baseline characteristics are displayed in Table 2. There were 7 males and 4 females in the control group and 5 males and 5 females in the intervention group. Median age was 64 in the control group and 66 in the intervention group. Mean APACHE II score was 16 in the control group and 17 in the intervention group. 17 patients were neurosurgical patients, 2 were general surgery patients and 2 were orthopaedic patients.

**Table 2:** Baseline characteristics between the control and intervention groups

	Control (*n* = 11)	Intervention (*n* = 10)
Age (years)	64 (60–69)	66 (61.5–71.5)
Male Gender	7 (63.6)	5 (50.0)
Weight (kg)	64.4 (57–79)	60 (52.8–63.4)
APACHE II	16 (12-18)	17 (15.3–20.8)
Surgery Category		
Neurosurgical	10 (90.9)	7 (70.0)
Surgical	1 (9.1)	3 (30.0)
Race		
Chinese	7 (63.6)	8 (80.0)
Malay	2 (18.2)	1 (10.0)
Indian	2 (18.2)	1 (10.0)
Pre-existing diabetes	4 (36.4)	5 (50.0)

Results presented as either n (%) or median (IQR)

Median calories achieved was 24.5 kcal per kg per day (IQR 22.3–25.3) in the control group and 23.3 kcal per kg per day (IQR 21.0–26.5) in the intervention group. Median protein fed was 1.2 g per kg per day (IQR 1.1–1.4) in the control and 1.6 g per kg per day (IQR 1.3–1.9) in the intervention group. Cycle ergometry was applied to patients in the intervention group for a median of 60 min per day and a patient had a median of 8.5 sessions in 14 days.

Measured cross sectional area decreased by a median of 4.7cm^2^ in the right rectus femoris in the control group and 1.8cm^2^ in the intervention group (*p* = 0.19), while the left rectus femoris cross sectional area decreased by 1.9cm^2^ in the control group and 0.1cm^2^ in the intervention group (*p* = 0.51) (Table 3).

**Table 3:** Decrease in measured cross sectional area of the rectus femoris in the control and intervention groups

	Control	Intervention	P-value
Right rectus femoris	4.7cm^2^	1.8cm^2^	*p* = 0.19
Left rectus femoris	1.9cm^2^	0.1cm^2^	*p* = 0.51

Duration of ventilation and use of indirect calorimetry was 2 to 13 days in the intervention group.

In the control group with use of indirect calorimetry, using a standard formula would have resulted in a median calorie deficit of 7 kcal, which is not statistically significant. However, the variance is large (from underfeeding 3072 kcal to over feeding 1622 kcal).

With regards to tolerance of feeding, daily gastric residual volume aspirated was not significantly different between the groups. However, although daily minimum and maximum blood sugar level measured is not significantly different, the insulin requirement for the intervention group was much higher. This may be confounded by the fact that there are more diabetics in the intervention group.

Other secondary outcomes measured for ICU and hospital lengths of stay, and mortality were not statistically significant (Table 4).

**Table 4:** Secondary outcomes

	Control (*n* = 11)	Intervention (*n* = 10)	P-value
ICU LOS	9 (5-13)	10 (5.5–22)	0.835
Hospital LOS	45 (26–59)	60 (40–147.5)	0.279
Mortality	3 (27.3)	3 (30.0)	> 0.999
Daily aspirates (ml)	37.7 (17.4–151.0)	51.5 (13.8–169.3)	0.835
Daily Min *BSL	8.3 (7.6–9.4)	7.85 (6.6–8.6)	> 0.999
Daily Max BSL	11.15 (9.5–11.8)	10.5 (8.9–1.45)	0.371
Daily insulin	7.4 (0.3–14.4)	26.5 (10.2–40.6)	0.027

Results presented as either n (%) or median (IQR)

*Blood sugar level

Functional outcome measures were attempted but due to poor conscious levels of the patients, the majority of the patients were not able to participate in the measurements.

## Discussion

In this pilot study, we found a trend towards decreased muscle loss in bilateral rectus femoris with our combined intervention of indirect calorimetry directed protocolised feeding and cycle ergometry. However, it did not reach statistical significance likely due to small numbers. We noted also the difference in decrease in muscle loss between the right and left rectus femoris muscles which could be because within our neurosurgical patients, we had more patients with right sided weakness as a result of their pathology. Prior studies done on indirect calorimetry directed feeding have not shown a significant impact on functional outcome [8], while studies have shown that physical therapy in the critically ill improves functional outcome only marginally or showed no difference in global muscle strength [9]. Kayambu et al. showed that early physical rehabilitation using a combination of muscle electrical stimulation and mobilization including cycling improved self-reported physical function but did not improve muscle strength and exercise capacity [10]. A study done by Machado et al. demonstrated that passive cycling exercise in combination with conventional physical therapy increased muscle strength but showed no difference in terms of duration of mechanical ventilation and length of hospital stay [11]. Kagan et al. investigated the effect of protein enriched enteral nutrition and early cycle ergometry on duration of mechanical ventilation, which showed no significant difference [12]. To our knowledge, there are no existing large randomised controlled trials comparing the use of indirect calorimetry directed feeding and cycle ergometry with empirical weight based feeding and standard physical therapy.

### Study strengths

This is a novel pilot study examining the combined effects of these 2 interventions on muscle wastage. These interventions may possibly have a symbiotic effect in reducing muscle wastage.

We initiated physical therapy at 1–2 days of ICU admission, even earlier than some studies in septic shock [16], and did not encounter any safety issues or premature termination of the cycle ergometry.

Through this pilot study, we have found that our interventions were feasible. Our patients who received indirect calorimetry directed feeding did not experience issues with feed intolerance except for 1 patient who was admitted to hospital for bowel surgery and subsequently aspirated and hence got admitted into our SICU.

Although our cycle ergometry machines are not designed specifically for the Asian habitus, however, we did not have difficulty fitting our patients to the cycle ergometry machine. Patients who were unable to cooperate managed to receive passive cycling. There were no injuries from the cycling.

This study was conducted in a predominantly neurosurgical patient population where mobilization out of bed is usually difficult and hence our interventions are a potential way to address the issue of muscle wastage.

### Study limitations

Limitations include our small sample size arising from difficulty in recruitment of patients due to our inclusion criteria and nature of our SICU patient population. Our unit receives many post elective surgery patients who were usually extubated quickly. We also received trauma patients who cannot receive cycle ergometry due to injuries. Also, many patients post emergency abdominal surgery have delayed initiation of enteral feeds. Hence, most of our patients are neurosurgical patients who have been admitted for intracranial bleeds and subarachnoid haemorrhages. However, neurosurgical patients with such pathology in particular usually are unable to mobilize out of bed for a prolonged period during recovery and hence our interventions may be particularly relevant to them. The small sample size unfortunately resulted in statistically insignificant results although there was a trend towards reduced muscle mass loss in the intervention group.

Another limitation is that we were only able to apply indirect calorimetry directed feeding to patients when they were intubated. After extubation, while in ICU, the patients were fed based on their last REE reading and daily caloric expenditure from cycling. After being discharged to the high dependency, dieticians subsequently planned their feeding regimen. This is a limitation as the indirect calorimetry directed feeding protocol may need to be applied over a longer duration in order to have a more significant effect. This is however a reflection of current practice in our institution which our study accommodated.

Also, the control and intervention groups did not have significantly different calorie intake per body weight. Although the protein intake in the intervention group was higher at 1.6 g per kg per day, the control group also had a satisfactory protein intake at 1.2 g per kg per day. This is within the ESPEN guidelines of 1.2 to 1.5 g per kg per day [6]. These may have contributed to the little difference in outcome measures.

Based on the latest 2018 European Society for Clinical Nutrition and Metabolism (ESPEN) guidelines, every critically ill patient staying for more than 48 h in the ICU should be considered at risk for malnutrition [6]. In the absence of a validated nutritional screening tool for critically ill patients, an individualized approach of measuring the energy requirements of each patient with indirect calorimetry, which may vary day to day due to clinical condition would be ideal. This has been recommended by both ESPEN and ASPEN (American Society for Parenteral and Enteral Nutrition Clinical guidelines). Therefore, if resources permit, critically ill patients, especially those with nutritional deficiency risk factors, should have indirect calorimetry measured. To prevent feed intolerance, we have a unit protocol of starting feeds at 20 ml per hour and checking gastric residual volume 4 hourly. The feed volume is increased by 20 ml per hour if the gastric residual volume is less than 250 ml. This serves to decrease incidence of feed intolerance and also to ramp up the feeds gradually such that overfeeding does not occur although the final target was 100% of the resting energy expenditure based on the indirect calorimetry. This protocol in addition to feed disruption for procedures and assessment for extubation translates to the recommended hypocaloric feeding in the early days of feed initiation.

Mitchell et al. compared changes in muscle mass as a factor of global changes in body composition with age. They reported a 0.5–1.0% loss of muscle mass per year after 70 years of age [17]. Muscle strength is an important factor to reduce frailty in the elderly and muscle strength increases with training. This can lead to improvements in functional mobility [18]. With the spotlight on post intensive care syndrome in the recent decade, perhaps a future study involving the elderly population can be done with a longer longitudinal follow up to delineate the effects of an optimized nutrition therapy with exercise on their frailty post ICU discharge.

In conclusion, we found a trend towards decreased muscle loss in bilateral rectus femoris with our combined intervention of indirect calorimetry directed protocolised feeding and cycle ergometry. However, it did not reach statistical significance likely due to small numbers. Our pilot study has shown that the interventions are feasible and this paves the way for a future larger study to be conducted.

## Data Availability

The datasets used during the study are available from the corresponding author on reasonable request.
